# Identifying the function of genes involved in excreted vesicle formation in *Acanthamoeba castellanii* containing *Legionella pneumophila*

**DOI:** 10.1186/s13071-023-05824-y

**Published:** 2023-06-28

**Authors:** Min-Jeong Kim, Eun-Kyung Moon, Hye-Jeong Jo, Fu-Shi Quan, Hyun-Hee Kong

**Affiliations:** 1 Department of Biomedical Science, Graduate School, Kyung Hee University, Seoul, Republic of Korea; 2 Department of Medical Zoology, Kyung Hee University School of Medicine, Seoul, Republic of Korea; 3 Medical Research Center for Bioreaction to Reactive Oxygen Species and Biomedical Science Institute, School of Medicine, Graduate school, Kyung Hee University, Seoul, Republic of Korea; 4 Department of Parasitology, Dong-A University College of Medicine, Busan, Republic of Korea

**Keywords:** *Acanthamoeba castellanii*, *Legionella pneumophila*, Excreted vesicles

## Abstract

**Background:**

*Legionella* spp. can survive and replicate inside host cells such as protozoa and macrophages. After enough growth, *Legionella* is released from the host cells as free legionellae or *Legionella*-filled vesicles. The vesicles support *Legionella* to survive for a long time in the environment and transmit to a new host. In this study, we identified the differentially expressed genes of *Acanthamoeba* infected by *Legionella* (ACA1_114460, ACA1_091500, and ACA1_362260) and examined their roles in the formation of the excreted vesicles and escape of *Legionella* from the *Acanthamoeba*.

**Methods:**

After ingestion of *Escherichia coli* and *Legionella pneumophila*, expression levels of target genes in *Acanthamoeba* were measured by real-time polymerase chain reaction (PCR) analysis. The roles of target genes were investigated by transfection of small interfering RNA (siRNA). The formation of *Legionella*-containing excreted vesicles and the vesicular co-localization with the lysosomes were examined by Giemsa stain and LysoTracker stain.

**Results:**

ACA1_114460, ACA1_091500, and ACA1_362260 were upregulated after ingestion of *Legionella* in *Acanthamoeba*. ACA1_114460- and ACA1_091500-silenced *Acanthamoeba* failed to form the *Legionella*-containing excreted vesicles. *Legionella* was released as free legionellae from the *Acanthamoeba*. When the ACA1_362260 of *Acanthamoeba* was silenced, *Legionella*-containing excreted vesicles were fused with the lysosome.

**Conclusions:**

These results indicated that ACA1_114460, ACA1_091500, and ACA1_362260 of *Acanthamoeba* played important roles in the formation of *Legionella*-containing excreted vesicles and inhibition of the lysosomal co-localization with the phagosome.

**Graphical Abstract:**

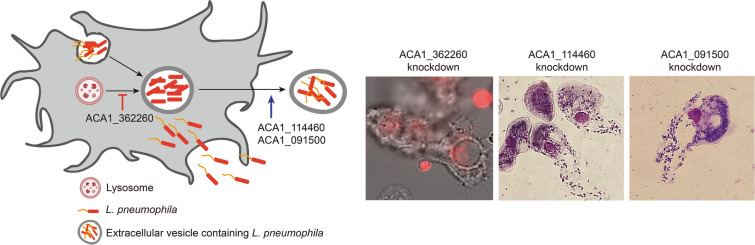

## Background

Given the ubiquitous nature of *Acanthamoeba*, humans are prone to contacting this protozoan organism, which consequently leads to various amoeba-borne ocular diseases [1–3]. While *Acanthamoeba* ingests various microorganisms in the surrounding environment to promote its growth in the trophozoite stage, these trophozoites are transformed into highly drug-resistant cysts under unfavorable conditions [4, 5]. The propensity of this organism to encyst under growth-limiting conditions is of importance, as this contributes to the persistence of pathogenic intracellular bacteria such as *Legionella pneumophila* [6, 7]. *Legionella pneumophila* is a gram-negative bacillus prevalent in the aquatic environment and its infection causes severe respiratory diseases including pneumonia and Legionnaires’ disease in humans [8]. Because *L. pneumophila* can thrive within eukaryotic host cells including *Acanthamoeba*, irrespective of trophozoites or cyst stages, it serves as a suitable host organism for *L. pneumophila*. Furthermore, identifying factors that regulate intracellular bacterial growth in *Acanthamoeba* will contribute to developing a novel treatment option for diseases inflicted by *L. pneumophila*.

Unlike bacteria-containing phagosomes, *L. pneumophila* forms a *Legionella*-containing vacuole (LCV) upon successful host cell infiltration to prevent its digestion via lysosomal fusion [9, 10]. The formation of this specialized vacuole supports intracellular replication in the host cells and enhances the virulence of *Legionella* [10, 11]. Inhibiting the phagosome–lysosome fusion that occurs during *L. pneumophila* infection requires a functional intracellular multiplication/defective in organelle trafficking (*icm*/*dot*) type-IV secretion system [12–14]. *Legionella pneumophila* is capable of replicating in host cells, which subsequently release free legionellae or *Legionella*-filled vesicles that aid in bacterial transmission [6]. The release of *Legionella*-containing vesicles to the surrounding environment may be necessary for surviving for a long time and efficient transmission to other host cells. As such, investigating how *L. pneumophila* forms these vesicles within *Acanthamoeba* spp. or their egress from hosts would be beneficial to understanding their replication and transmission.

The molecular pathogenesis of *L. pneumophila* infection in *Acanthamoeba* and several other amoebic organisms are similar to those of macrophages, and as such, these protozoans were used as good models to investigate *Legionella*–macrophage interactions [10, 14, 15]. However, *L. pneumophila* grown in human monocytes and *Acanthamoeba* had different gene-expression profiles, thus suggesting that survival strategies employed by *L. pneumophila* may vary across hosts. For example, when *L. pneumophila* was cultured in THP-1 cells, expression of the pyroptotic protein *flaA* was downregulated and the pyroptosis-inhibiting *sdhA* protein expression which contributes to LCV stabilization was upregulated. Contrary to this finding, when *L. pneumophila* was permitted to grow within *Acanthamoeba castellanii*, *sdhA* gene expression was downregulated [16]. Numerous studies have delineated several factors involved in *L. pneumophila* vesicle formation or their release from *Acanthamoeba*. After *Legionella* has replicated enough in specialized vesicles, it is released via an exocytic pathway in protozoa that is either absent or unused in mammalian cells [17]. It has been demonstrated that *Legionella* transmission activator (LetA) was necessary for intracellular multiplication in *A. castellanii*, and letA mutants exhibited reduced infectivity [18]. The carboxy terminus of *Legionella* isoprenylcysteine carboxyl methyltransferase (IcmT) is essential for pore formation involved in bacterial egress, whereas its amino terminus is essential for the export of specific effectors [19, 20]. *Legionella* effector proteins, LepA and LepB, played a role in the non-lytic release of *L. pneumophila* from *A. castellanii* [21]. In addition to the aforementioned effectors, the genes of the *icm/dot* type-IV secretion system of *Legionella* required for LCV formation and multiplication in the host cells are well known. Nonetheless, as demonstrated above, gene expression-related studies involving *L. pneumophila* and *Acanthamoeba* spp. were predominantly focused on the former of the two. Specifically, *Acanthamoeba*-related factors contributing to the growth and release of *Legionella* remain largely elusive.

To address these limitations, in our previous study, we conducted a comparative analysis of differentially expressed genes (DEGs) in *A. castellanii* after ingesting either *Escherichia coli* or *L. pneumophila* [22]. Among these DEGs, 502 genes were upregulated in *Acanthamoeba* infected by *Legionella*. In the aforementioned study, we identified 22 genes involved in vesicle trafficking, membrane fusion, and phagocytosis. Interestingly, several of the 22 genes described above were upregulated in *Legionella*-infected *Acanthamoeba* but not when *E. coli* was taken up by the *Acanthamoeba*. Therefore, we hypothesized that these genes could be involved in vesicle formation which promotes *L. pneumophila* survival. To address this, in this study, we selected these upregulated genes in *Legionella*-infected *Acanthamoeba* and investigated their function in vesicle formation as well as their release from *Acanthamoeba*. Also, to account for *L. pneumophila* and host relationship issues, we used both environmental and clinical isolates of *Acanthamoeba*.

## Methods

### Cell culture and infection of bacteria

*Acanthamoeba castellanii* Castellani (ATCC 30868, clinical isolate) and *Acanthamoeba castellanii* Neff (ATCC 30011, environmental isolate) were obtained from the American Type Culture Collection and cultured axenically in peptone-yeast-glucose (PYG) medium at 25 °C. *Legionella pneumophila* Philadelphia-1 (ATCC 33152) was cultured on a buffered charcoal yeast extract (BCYE) agar plate at 37 °C with 5% CO_2_. *Escherichia coli* DH5α (Enzynomics, Seoul, Republic of Korea) was cultured in tryptone-yeast-NaCl lysogeny broth (LB) media at 37 °C using a shaking incubator. *Acanthamoeba castellanii* was infected by *L. pneumophila* at multiplicity of infection (MOI) of 100 as previously described [16]. Briefly, *L. pneumophila* was diluted in phosphate-buffered saline (PBS) until the optical density (OD)_600_ absorbance reading reached 1, which corresponds to 10^9^ colony-forming units (CFU)/ml [23]. Next, 1 × 10^7^ of *Acanthamoeba* were incubated with 1 ml of *Legionella* suspension at 37 °C with 5% CO_2_ for 1 h in PYG medium. After incubation, *Acanthamoeba* was washed with Page’s Amoeba Saline (PAS) and incubated with new PYG media containing 100 μg/ml of gentamicin for 2 h to kill extracellular *Legionella*. *Acanthamoeba* infected with *Legionella* (A+L) was washed with PAS twice and incubated with new PYG media for 12 h in a 25 °C incubator. *Acanthamoeba* infection of *E. coli* (A+E) was conducted in the same way.

### *Gene expression analysis via real-time polymerase chain reaction (PCR)*

The expression of target genes (Table 1) was determined by real-time PCR analysis. The total RNA was purified using an RNeasy Mini Kit (Qiagen, Hilden, Germany), and the complementary DNA (cDNA) was synthesized using a RevertAid First Strand cDNA Synthesis Kit (Thermo Fisher Scientific, Waltham, MA, USA) following the manufacturer’s instructions. Real-time PCR was conducted using a Magnetic Induction Cycler PCR machine (PhileKorea, Seoul, Republic of Korea) as previously described [22]: pre-incubation at 95 °C for 1 min, followed by 40 cycles of 95 °C for 15 s and 60 °C for 30 s. All reaction mixtures were made using a Luna Universal quantitative PCR (qPCR) Master Mix (New England Biolabs, Ipswich, MA, USA) with different sense and antisense primers (Table 2).

**Table 1:** Differentially expressed genes in *Acanthamoeba* during phagocytosis of *E. coli* and *L. pneumophila* by RNA sequencing analysis

No.	Gene symbol	A+E/A^a^	A+L/A^b^	A+L/A+E^c^	Product
1	ACA1_114460	4.361	7.172	1.645	SNARE domain-containing protein
2	ACA1_091500	1.015	3.849	3.792	R-SNARE, VAMP72-family protein
3	ACA1_265950	0.266	5.001	18.801	Golgi family protein (ER vesicle transporter)
4	ACA1_362260	192.089	4.079	0.021	Hypothetical protein
5	ACA1_328910	16.325	1099.734	67.365	Hypothetical protein
6	ACA1_096640	0.707	20.427	28.892	Hypothetical protein

^a^A+E/A: *A. castellanii* which ingested *E. coli*/*A. castellanii*

^b^A+L/A: *A. castellanii* which ingested *L. pneumophila*/*A. castellanii*

^c^A+L/A+E: *A. castellanii* which ingested *L. pneumophila*/*A. castellanii* which ingested *E. coli*

**Table 2:** Primer sequences for real-time PCR

No	Gene symbol	Product	Primer sequence (5′ → 3′)
1	ACA1_114460	SNARE domain-containing protein	F: TTCATGCAGACCTTCACCAG
			R: GGTGGTGTCGACCTTGTTCT
2	ACA1_091500	R-SNARE, VAMP72-family protein	F: GCAAGATCGAGGAGATGGTC
			R: ACGGTCCAGAACAGGTTACG
3	ACA1_265950	Golgi family protein	F: ATGCTCATCAGCTGGGAAGT
			R: TGGGACTGTGACGTTGATGT
4	ACA1_362260	Hypothetical protein	F: CTTCTTCATCGTCGTCGTCA
			R: CCACCCAGTTGGAGTAGTCG
5	ACA1_328910	Hypothetical protein	F: AACCTTGGCATCACCAACTC
			R: TCAGCTGTCTGGTGATGAGG
6	ACA1_096640	Hypothetical protein	F: ACCAAGCTGCTCTTTGTCGT
			R: GTCACGGTAGTAGGCCTTGC

### Gene silencing

Small interfering RNAs (siRNAs) targeting ACA1_114460, ACA1_091500, and ACA1_362260 of *Acanthamoeba* were synthesized by Bioneer, Inc. (Bioneer, Daejeon, Republic of Korea), based on their cDNA sequences (Table 3). The siRNA (final concentration of 100 nM) was transfected into live *Acanthamoeba* trophozoites at a cell density of 4 × 10^5^ cells using the Effectene transfection reagent (Qiagen, Hilden, Germany) following the manufacturer’s protocol. The transfection efficiency of siRNA was determined by fluorescing cells under a fluorescent microscope (Leica, Wetzlar, Germany).

**Table 3:** siRNA sequences used in this study

No	Gene symbol	Target gene	Target sequence (5′ → 3′)
1	ACA1_114460	SNARE domain-containing protein	F: CACUUCAUGCAGACCUUC
			R: UGAAGGUCUGCAUGAAGU
2	ACA1_091500	R-SNARE, VAMP72-family protein	F: GCAAGAUCGAGGAGAUGGU
			R: ACCAUCUCCUCGAUCUUGC
3	ACA1_362260	Hypothetical protein	F: CCUGCACUUUCCCAUUCC
			R: UGGAAUGGGAAAGUGCAG

### Observation of excreted vesicles

Excreted vesicles of *A. castellanii* containing *L. pneumophila* were observed with Giemsa and LysoTracker staining. *Acanthamoeba* was transfected with siRNA (siRNA-A) and infected by *Legionella* (siRNA-A+L) as mentioned above. For the Giemsa stain, cells were fixed with methanol for 5 min and stained with Giemsa solution (Sigma Aldrich, Burlington, MA, USA) for 10 min. For the LysoTracker stain, cells were stained with 50 μM LysoTracker Red DND-99 (Invitrogen, Carlsbad, CA, USA) for 1 h. Stained cells were observed under a fluorescent microscope. Vesicles or phagolysosomes were counted from a total of three randomly selected fields of view under the microscope at 1000× magnification.

### Statistical analysis

Data are presented as mean ± standard deviation (SD) from three independent experiments. Student’s *t*-tests were performed using GraphPad Prism version 8 (Dotmatics, San Diego, CA, USA). Statistical significance between the means of groups was denoted using an asterisk. *P*-values less than 0.05 was considered statistically significant (*P < 0.05, **P < 0.01, ***P < 0.001, and ****P < 0.0001).

## Results

### Identification of upregulated genes in *Legionella*-infected *Acanthamoeba*

**Fig. 1:**
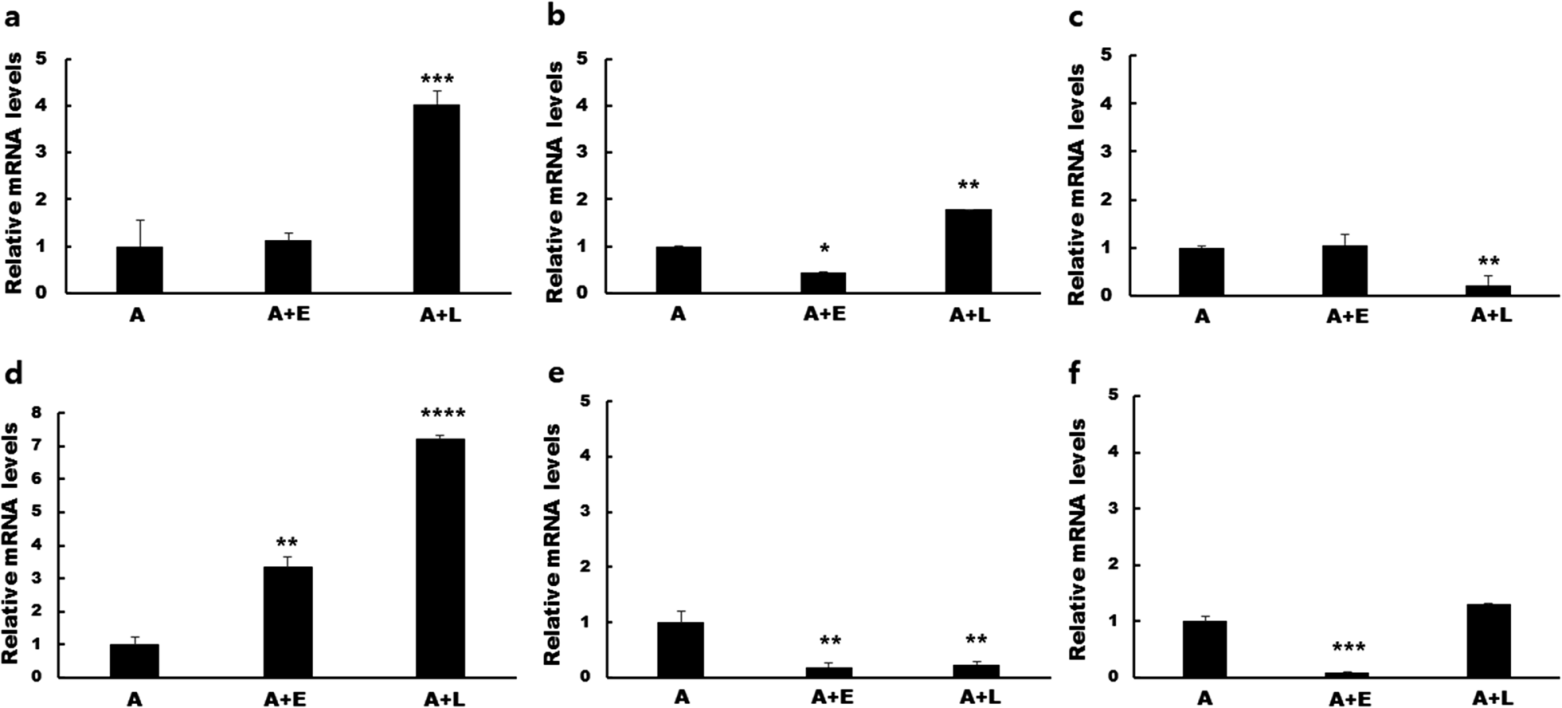
Real-time PCR analysis of differentially expressed genes in *Acanthamoeba* during phagocytosis of *E. coli* and *L. pneumophila*. *Acanthamoeba castellanii*-ingested *E. coli* and *L. pneumophila* for 12 h and differential gene expression of six genes (**a** ACA1_114460, **b** ACA1_091500, **c** ACA1_265950, **d** ACA1_362260, **e** ACA1_328910, and **f** ACA1_096640) were determined by real-time PCR. The transcriptional levels of six genes in *Acanthamoeba* (A) were compared to those of *Escherichia*-ingested *Acanthamoeba* (A+E) and *Legionella*-ingested *Acanthamoeba* (A+L). Data are expressed as mean ± SD of three separate experiments. Asterisks denote statistically significant differences (* *P* < 0.05, ** *P* < 0.01, *** *P* < 0.001, and **** *P* < 0.0001 compared to *Acanthamoeba* control)

Previously, we performed RNA sequencing to identify DEGs in *A. castellanii* that phagocytosed either *E. coli* or *L. pneumophila* [22]. Among these DEGs, we identified a total of six DEGs that were upregulated in *A. castellanii* Castellani post-*L. pneumophila* ingestion (A+L/A), which were associated with phagosomal maturation (Table 1). Based on the National Center for Biotechnology Information (NCBI) Basic Local Alignment Search Tool (BLAST) search results, ACA1_114460, ACA1_091500, and ACA1_265950 showed sequence similarity with SNARE domain-containing protein, R-SNARE VAMP72-family protein, and Golgi family protein, each respectively. ACA1_362260, ACA1_328910, and ACA1_096640 were identified as hypothetical proteins. To confirm their expression levels, cDNA from *A. castellanii* Castellani and *A. castellanii* Neff which ingested either *E. coli* (A+E) or *L. pneumophila* (A+L) were acquired and real-time PCR was performed using the gene-specific primers listed below (Table 2). Of the six genes, the expressions of only three genes (ACA1_114460, ACA1_091500, and ACA1_362260) from the A+L were induced to significantly higher levels than A+E controls (Fig. 1). Specifically, the ACA1_114460 gene was upregulated by approximately fourfold in A+L whereas changes were negligible for the A+E group (Fig. 1a). *Escherichia coli* ingestion resulted in the significant downregulation of ACA1_091500 compared to *A. castellanii* control while their expression was significantly upregulated in A+L (Fig. 1b). ACA1_265950 expressions were unchanged in A+E, though substantial inhibition was observed in the A+L (Fig. 1c). Ingestion of bacteria, irrespective of *E. coli* or *L. pneumophila*, enhanced the gene expression of ACA1_362260, with expression reaching a sevenfold increase in the A+L group (Fig. 1d). Conversely, ACA1_328910 gene expressions were suppressed in both A+E and A+L groups (Fig. 1e). Phagocytosing *E. coli* suppressed ACA1_096640 mRNA levels but their expressions remained negligibly changed in the A+L (Fig. 1f).

### siRNA-mediated gene silencing

**Fig. 2:**
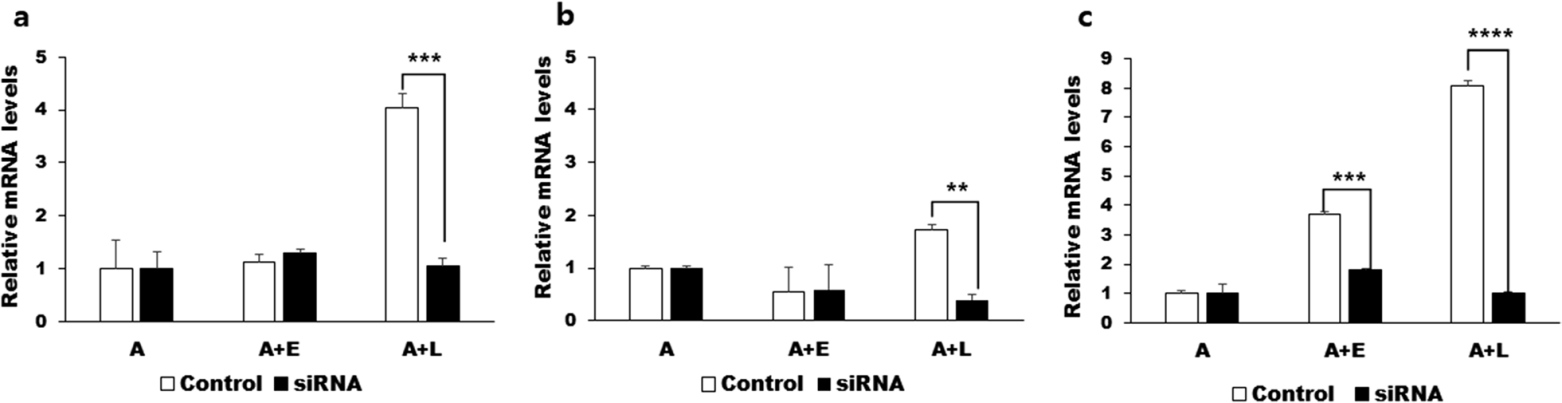
Real-time PCR analysis after transfection of siRNA. *Acanthamoeba castellanii* were transfected with siRNA designed against ACA1_114460, ACA1_091500, and ACA1_362260 prior to bacterial ingestion for 12 h. Knockdown of gene expression was confirmed by real-time PCR (**a** ACA1_114460, **b** ACA1_091500, and **c** ACA1_362260). Data are expressed as mean ± SD of three separate experiments. Asterisks denote statistically significant differences (** *P* < 0.01, *** *P* < 0.001, and **** *P* < 0.0001) between the control group and the siRNA-transfected group

To observe the effects of gene knockdown in vitro, three siRNAs specific for ACA1_114460, ACA1_091500, and ACA1_362260 were prepared (Table 3). After transfecting *Acanthamoeba* with the three siRNAs, real-time PCR was performed to verify the gene silencing efficiency. Transfecting ACA1_114460-specific siRNA did not incur any changes to mRNA levels in the A or A+E control groups. However, in the A+L group, siRNA transfection significantly suppressed the ACA1_114460 mRNA expression to basal levels (Fig. 2a). Similar findings were observed for ACA1_091500. Transfection of siRNA specific to the ACA1_091500 gene had a negligible effect on A and A+E, but drastically reduced their expression in the A+L experimental group (Fig. 2b). Interestingly, unlike the other two genes, ACA1_362260 siRNA transfection resulted in the inhibition of specific mRNA expression in both A+E and A+L groups (Fig. 2c).

### Effect of gene silencing on the excreted vesicle formation

**Fig. 3:**
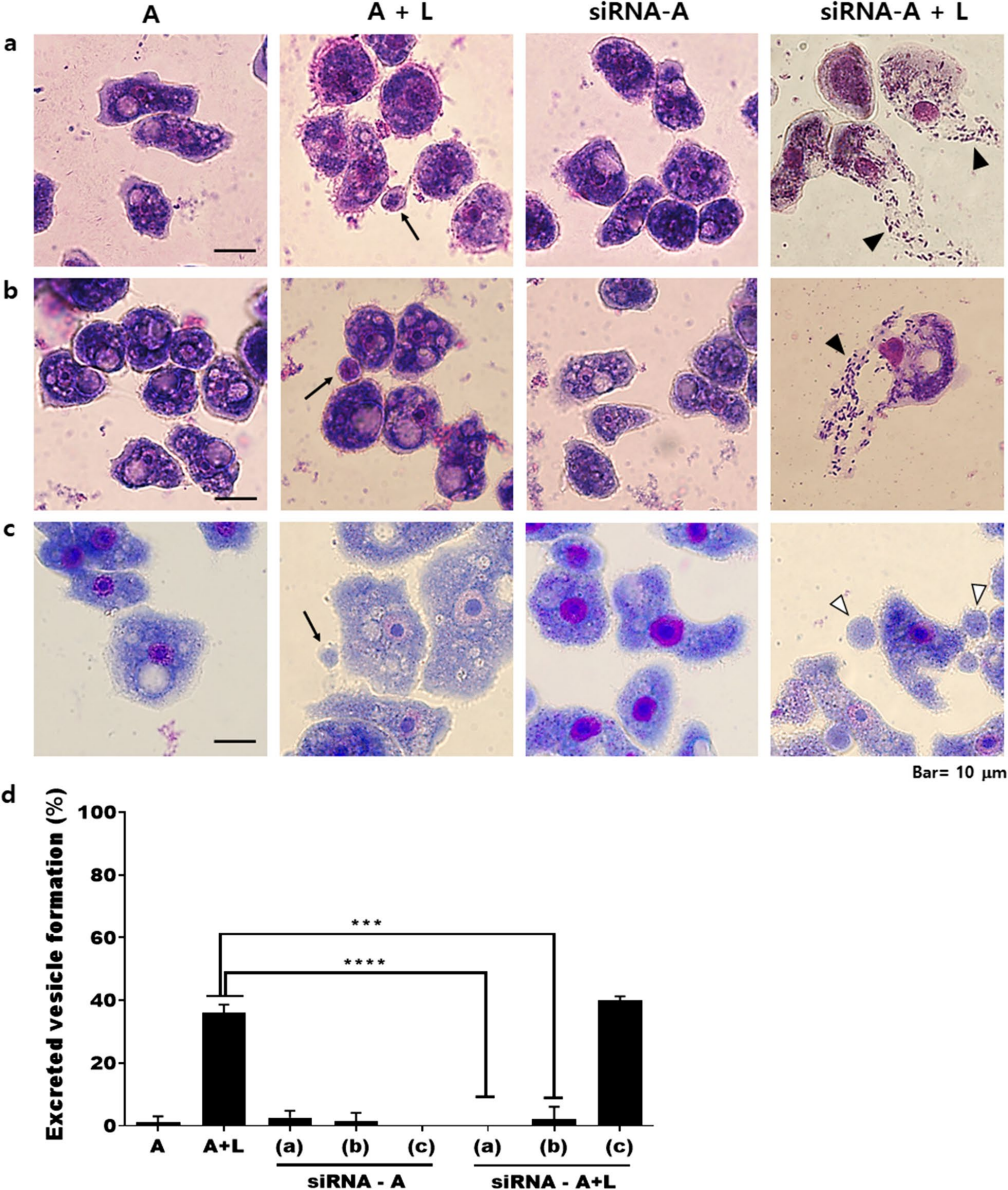
*Legionella* in siRNA-transfected *Acanthamoeba*. siRNA-transfected *A. castellanii* which ingested *L. pneumophila* for 12 h (siRNA-A+L). Cells were stained with Giemsa staining solution. **a** ACA1_114460 siRNA-transfected *A. castellanii* Castellani, **b** ACA1_091500 siRNA-transfected *A. castellanii* Castellani, and **c** ACA1_362260 siRNA-transfected *A. castellanii* Neff. **d** Quantification of excreted vesicles following siRNA treatment. Arrows: excreted vesicles containing *Legionella*, black arrowheads: free legionellae, and white arrowheads: excreted vesicles containing *Legionella*. **A** *Acanthamoeba* only, **A+L** *Acanthamoeba* with *L. pneumophila*, **siRNA-A** siRNA-transfected *Acanthamoeba*, and **siRNA-A+L** siRNA-transfected *Acanthamoeba* co-cultured with *L. pneumophila*. Images were acquired at ×1000 magnification. Asterisks denote statistical significance compared to A+L control (*** *P* < 0.001, **** *P* < 0.0001)

To investigate the effect of ACA1_114460, ACA1_091500, and ACA1_362260 gene knockdown in *Acanthamoeba*, Giemsa staining was conducted followed by microscopic observations. After 12 h of *Legionella* infection, *Acanthamoeba* (A+L) produced excreted vesicles containing *Legionella*, as indicated by the black arrows in the A+L panel (Fig. 3). However, siRNA treatment of ACA1_114460 and ACA1_091500 prevented the formation of these vesicles and resulted in the dispersion of legionellae after bursting from the *Acanthamoeba* host, as shown in the siRNA-A+L panel (Fig. 3a, b). The black arrowheads indicate the extracellular release of *L. pneumophila* from *Acanthamoeba*. On the contrary, ACA1_362260 siRNA-transfected *Acanthamoeba* did not affect the formation of these excreted vesicles, as indicated by their intact structures denoted by white arrowheads (Fig. 3c). No noticeable changes were observed in *A. castellanii* even after siRNA transfection (siRNA-A column). Excreted vesicles from all groups were quantified under the microscope (Fig. 3d). In A+L, approximately 40% vesicle formation was observed. This phenomenon, however, was not detected in ACA1_114460 and ACA1_091500 siRNA-transfected *Acanthamoeba*. The only exception to this was the ACA1_362260 siRNA-treated group, which demonstrated a similar level of vesicle formation to that of the control group (A+L).

### Effect of gene silencing on the phagolysosome

**Fig. 4:**
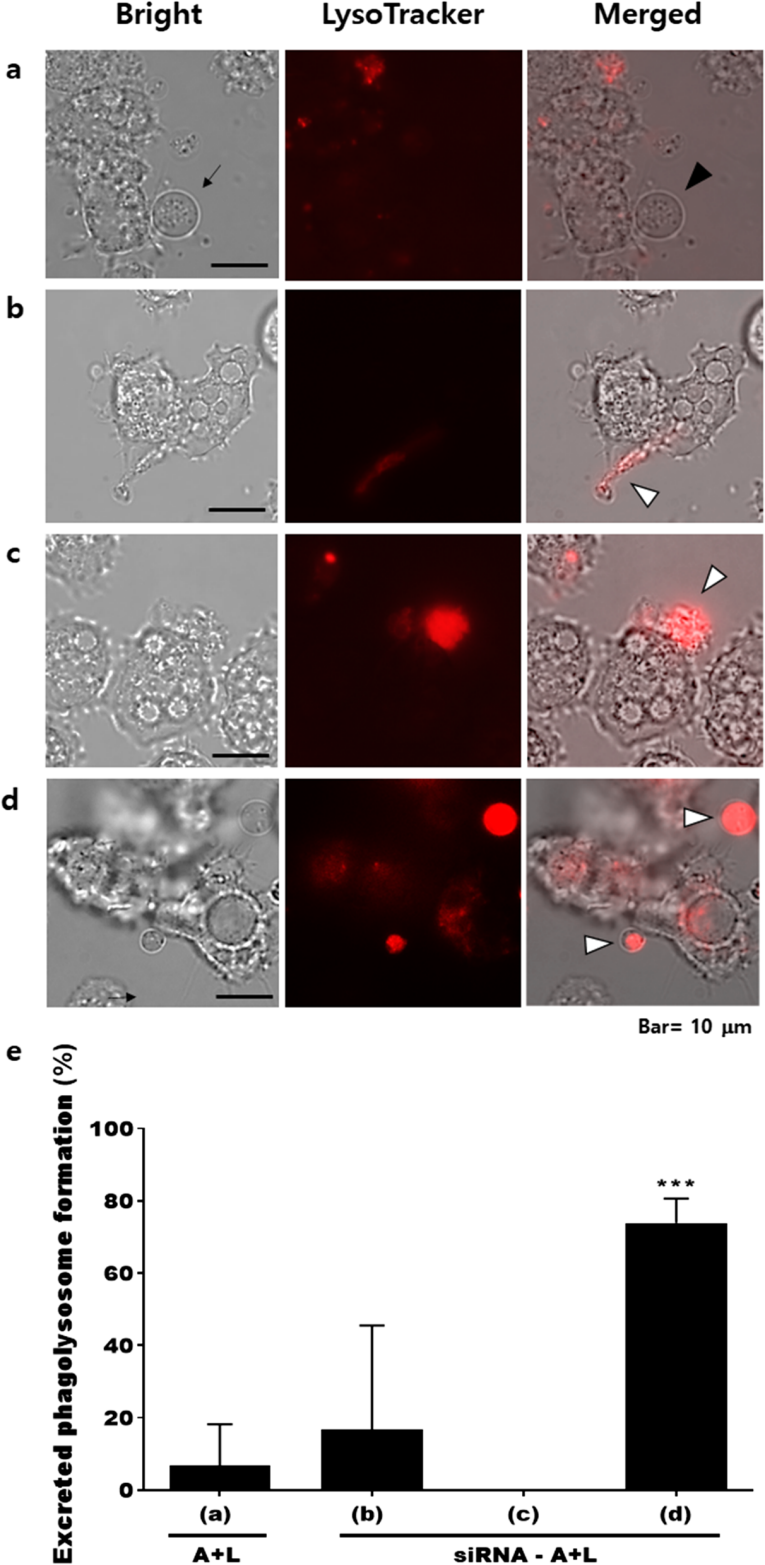
LysoTracker staining after transfection of siRNA. ACA1_114460, ACA1_091500, or ACA1_362260-specific siRNA was inoculated into *A. castellanii*, which were subsequently co-cultured with *L. pneumophila* for 12 h (siRNA-A+L). *Acanthamoeba* and excreted vesicles containing *Legionella* were stained with LysoTracker. **a** A+L, **b** ACA1_114460 siRNA-transfected A+L, **c** ACA1_091500 siRNA-transfected A+L, **d** ACA1_362260 siRNA-transfected A+L, **e** excreted phagolysosome quantification. Arrows, excreted vesicles containing *Legionella*; black arrowheads, excreted vesicles containing *Legionella* without lysosomes; and white arrowheads, excreted vesicles containing *Legionella* and lysosomes. Images were acquired at ×1000 magnification

To investigate the gene silencing effect of ACA1_362260 on the lysosomes localized within the excreted vesicles, LysoTracker staining, and microscopy were performed. The normal *Acanthamoeba* ingesting *Legionella* formed the excreted vesicles containing *Legionella*, as denoted by the black arrow (Fig. 4a). Lysosomes were not detected in the vesicles (black arrowhead, Fig. 4a). In *Acanthamoeba* transfected with either ACA1_114460 or ACA1_091500 siRNA, despite the vesicle excretion failure, partial staining with LysoTracker was observed (white arrowheads, Figs. 4b, c). Contrastingly, in the ACA1_362260 silenced *Acanthamoeba*, lysosome-containing excreted vesicles (white arrowheads, Fig. 4d) were separated from the *Acanthamoeba* host cell. The *Acanthamoeba* transfected with ACA1_362260 siRNA seems to have failed to inhibit phagolysosome formation. Excreted phagolysosomes were enumerated under the microscope (Fig. 4e). Consistent with the above findings, phagolysosomal formation was predominantly observed in ACA1_362260 siRNA-treated group, whereas ACA1_114460 or ACA1_091500 siRNA transfection resulted in negligible changes compared to the control group.

## Discussion

*Legionella pneumophila* is an intracellular pathogen that could replicate within eukaryotic host cells, and it can be transmitted to humans via aerosols containing infectious particles [6]. In this study, we investigated the role of several genes upregulated in *Acanthamoeba* following *L. pneumophila* ingestion. Our findings revealed that *L. pneumophila* replicated within *A. castellanii* and were released into the extracellular vicinity in a membrane-bound state. We also observed that siRNA targeting the ACA1_114460, ACA1_091500, and ACA1_362260 inhibited the formation of these excreted vesicles or lysosomal co-localization with the vesicles.

The DEGs ACA1_114460 and ACA1_091500 of *A. castellanii* were identified as a SNARE domain-containing protein and an R-SNARE VAMP72 family protein, respectively. Eukaryotic cells contain many internal organelles surrounded by membrane boundaries, and a large family of soluble *N*-ethylmaleimide-sensitive factor (NSF) attachment protein receptors (SNAREs) have been suggested to be involved in the mechanisms of vesicle trafficking, budding, and fusion [24–27]. SNAREs were functionally classified according to the membrane component associated with the vesicles (v-SNAREs) or the target compartment (t-SNAREs), and also could be structurally distinguished as Q-SNAREs and R-SNAREs based on the residue present in the center of the motif [28]. R-SNAREs can be subdivided into short vesicle-associated membrane proteins (VAMPs) and long VAMPs (longins), depending on whether they contain a short and variable domain or a conserved longin domain [28]. Given the nature of these SNAREs and their role in vesicle trafficking, we initially anticipated that knocking down these genes would affect *L. pneumophila* vesicle formation in *A. castellanii*. In line with this notion, our results confirmed that ACA1_114460 and ACA1_091500 of *A. castellanii* are involved in the formation of membrane-bound organelles (Fig. 3). ACA1_362260 of *A. castellanii* was identified as a hypothetical protein with an unknown function. Because transfection of ACA1_362260 siRNA did not inhibit vesicle formation, as was the case for ACA1_114460 and ACA1_091500, we reasoned that this hypothetical protein could be involved in a different intracellular vesicle-related process such as lysosome fusion. To ascertain this, *Acanthamoeba* ingesting *Legionella* was stained with LysoTracker post-siRNA treatment. Surprisingly, lysosomal conjugation of the vesicle was observed, and silencing the ACA1_362260 gene resulted in the release of lysosome-containing vesicles (Fig. 4d). Based on this observation, the ACA1_362260 gene is likely to be responsible for preventing lysosomal co-localization with the vesicles.

It is well known that *L. pneumophila* uses its *icm/dot* type-IV secretion system to translocate approximately 300 effector proteins into the host cell, which are required to establish the *Legionella*-containing vesicles necessary for its survival and replication [29–31]. Although earlier studies have revealed that these effectors of *Legionella* could control the vesicle trafficking of the host cell, the roles of effector-interacting proteins of the host cells remain unknown. Endosymbiont *L. pneumophila* within *A. castellanii* regulated the expression of numerous host genes including ACA1_114460, ACA1_091500, and ACA1_362260 [22]. Several interesting findings were reported from earlier studies, documenting the differences in *L. pneumophila* exocytic pathways between various organisms. For example, *L. pneumophila* induces apoptosis in macrophages and releases free-form *L. pneumophila* into the surroundings [32]. In *Acanthamoeba* spp., on the other hand, *L. pneumophila* can be released either as a free-living form or enclosed in spherical food vacuoles [4]. In support of this, our data shows that siRNA treatment inhibits *Legionella*-containing vacuole release. Based on this notion, we speculate that the VAMP and SNARE-like proteins that contributed to vacuole formation reported in our study are specific to *Acanthamoeba*. Though our findings did reveal that these genes are involved in *L. pneumophila*-containing vesicle formation and their release from *A. castellanii*, the exact function and mechanism of action for these genes remain unknown. Furthermore, in the case of ACA1_362260, the viability of legionellae contained within the lysosome-containing excreted vesicle needs to be confirmed. As such, more work is needed to identify effector molecules that control the function of these three proteins in addition to elucidating how these proteins affect *L. pneumophila* survival. These further studies investigating the molecular interactions will provide great information on the research of intracellular proliferation and transmission of *Legionella*.

## Conclusion

In summary, we identified three highly expressed genes (ACA1_114460, ACA1_091500, and ACA1_362260) in *A. castellanii* ingesting *L. pneumophila*. ACA1_114460 and ACA1_091500 were involved in the formation of excreted vesicles containing *Legionella*. ACA1_362260 was involved in inhibiting the lysosome co-localization with the *Legionella*-containing vesicle. These results provided important information for studying the survival strategy of *Legionella* in *Acanthamoeba* and the transmission of *Legionella* to another host.

## Data Availability

The dataset supporting the conclusion of this article is included within the article.

## Abbreviations

qPCR: Quantitative polymerase chain reaction
siRNA: Small interfering RNA
DEGs: Differentially expressed genes
CFU: Colony-forming unit
cDNA: Complementary DNA
SD: Standard deviation
A: *Acanthamoeba castellanii*
A+E: *Acanthamoeba castellanii* which ingested *Escherichia coli*
A+L: *Acanthamoeba castellanii* which ingested *Legionella pneumophila*
siRNA-A+L: siRNA-transfected *Acanthamoeba castellanii* which ingested *Legionella pneumophila*
